# 

**Published:** 1886-01

**Authors:** 


					﻿
JAN., 1886.
$1.00 per year.
ejoVRNAL^
111 \ri h
ESTABLISHED 1854
W33
1
OFFICE, 75 & 77 BARCLAY STREET, NEW YORK.
Entered as Second-class Matter at the Post-Office, New York.
				

